# Angiotensin Converting Enzyme Inhibitors versus Receptor Blockers in Patients with Ventricular Tachyarrhythmias

**DOI:** 10.3390/jcm11051460

**Published:** 2022-03-07

**Authors:** Tobias Schupp, Michael Behnes, Mohammad Abumayyaleh, Kathrin Weidner, Kambis Mashayekhi, Thomas Bertsch, Ibrahim Akin

**Affiliations:** 1 First Department of Medicine, University Medical Centre Mannheim (UMM), Faculty of Medicine Mannheim, University of Heidelberg, DZHK (German Center for Cardiovascular Research) Partner Site Heidelberg/Mannheim, Theodor-Kutzer-Ufer 1-3, 68167 Mannheim, Germany; tobias.schupp@umm.de (T.S.); mohammad.abumayyaleh@umm.de (M.A.); kathrin.weidner@umm.de (K.W.); ibrahim.akin@umm.de (I.A.); 2 Department of Cardiology and Angiology II, University Heart Center Freiburg, 79189 Bad Krozingen, Germany; kambis.mashayekhi@universitaets-herzzentrum.de; 3 Institute of Clinical Chemistry, Laboratory Medicine and Transfusion Medicine, Nuremberg General Hospital, Paracelsus Medical University, 90419 Nuremberg, Germany; thomas.bertsch@klinikum-nuernberg.de

**Keywords:** ventricular tachycardia, ventricular fibrillation, mortality, ACE inhibitor, ARB, medical treatment, pharmacological drugs

## Abstract

Data investigating the prognostic value of treatment with angiotensin converting enzyme inhibitors (ACEi) and receptor blockers (ARB) usually focusses on patients presenting with heart failure (HF) or acute myocardial infarction (AMI). However, by preventing adverse cardiac remodeling, ACEi/ARB may also decrease the risk of ventricular tachyarrhythmias and sudden cardiac death (SCD). Although ventricular tachyarrhythmias are associated with significant mortality and morbidity, only limited data are available focusing on the prognostic role of ACEi/ARB, when prescribed for secondary prevention of SCD. Therefore, this study comprehensively investigates the role of ACEi versus ARB in patients with ventricular tachyarrhythmias. A large retrospective registry was used including consecutive patients with episodes of ventricular tachycardia (VT) or fibrillation (VF) from 2002 to 2015. The primary prognostic outcome was all-cause mortality at three years, secondary endpoints comprised a composite arrhythmic endpoint (i.e., recurrences of ventricular tachyarrhythmias, ICD therapies and sudden cardiac death) and cardiac rehospitalization. A total of 1236 patients were included (15% treated with ARB and 85% with ACEi) and followed for a median of 4.0 years. At three years, ACEi and ARB were associated with comparable long-term mortality (20% vs. 17%; log rank *p* = 0.287; HR = 0.965; 95% CI 0.689–1.351; *p* = 0.835) and comparable risk of the composite arrhythmic endpoint (HR = 1.227; 95% CI 0.841–1.790; *p* = 0.288). In contrast, ACEi was associated with a decreased risk of cardiac rehospitalization at three years (HR = 0.690; 95% CI 0.490–0.971; *p* = 0.033). Within the propensity score matched cohort (i.e., 158 patients with ACEi and ARB), ACEi and ARB were associated with comparable long-term outcomes at three years. In conclusion, ACEi and ARB are associated with comparable risk of long-term outcomes in patients presenting with ventricular tachyarrhythmias.

## 1. Introduction

Angiotensin converting enzyme inhibitors (ACEi) and receptor blockers (ARB) were shown to reduce all-cause mortality and risk of sudden cardiac death (SCD) in patients with acute myocardial infarction (AMI) and heart failure (HF) with reduced left ventricular ejection fraction (i.e., LVEF ≤ 40%) when prescribed for primary prevention of SCD [1,2,3,4]. By promoting transforming growth factor beta-1-synthesis, angiotensin II may stimulate the formation of fibrosis tissue, which increases the risk of arrhythmogenesis due to facilitation of re-entry, especially in patients with ischemic heart disease. Furthermore, angiotensin II plays an important role as a vasoconstrictor. By increasing wall pressure and stretch, angiotensin II may also cause so-called electrical remodeling by prolonging conduction time and favoring conduction heterogeneity within cardiac myocytes. Moreover, angiotensin II was shown to have a direct effect on ion channels leading to increased calcium influx, which in turn favors the occurrence of atrial and ventricular tachyarrhythmias [5,6]. These pathophysiological aspects suggest decreased arrhythmic events in patients treated with ACEi/ARB. In line, decreased rates of SCD rates were observed within a large meta-analysis, including patients with AMI and HF [7]. However, Guideline recommendations for the prevention of ventricular tachyarrhythmias and SCD predominantly rely on patients treated with ACEi or ARB, who did not have prior episodes of ventricular tachyarrhythmias [6]. Therefore, within the current AHA/ACC/HRS Guidelines, ACEi/ARB have a class IA indication only in patients with LVEF ≤ 40% [8]. Using a large retrospective registry, we recently demonstrated that prescription of ACEi/ARB is associated with decreased all-cause mortality at three years in patients surviving index episodes of ventricular tachyarrhythmias, when prescribed for secondary prevention of SCD as compared to patients not treated with ACEi/ARB. However, prognosis of patients treated with ACEi was not compared to patients with ARB [9].

Accordingly, the risk of ventricular tachyarrhythmias in the presence or absence of ACEi/ARB therapy was merely investigated within rather small registries [10,11]. The GRACE study is one of the largest trials that investigated the risk of appropriate ICD shocks in the presence or absence of ACEi/ARB in patients with systolic HF and LVEF ≤ 35%, demonstrating reduced risk of ICD shocks at five years of follow-up [12]. However, data directly comparing the prognosis of patients treated with ACEi versus ARB are limited [13,14,15]. Therefore, the present study investigates the prognosis for patients with ventricular tachyarrhythmias treated with ACEi as compared to ARB on the primary endpoint of all-cause mortality, as well as on secondary endpoints (composite arrhythmic endpoint (i.e., recurrence of ventricular tachyarrhythmias, appropriate ICD therapies, SCD) and cardiac rehospitalization) at three years.

## 2. Materials and Methods

### 2.1. Data Collection and Documentation

The present study retrospectively included all patients surviving index episodes of ventricular tachyarrhythmias (i.e., ventricular tachycardia (VT) and ventricular fibrillation (VF)) on admission from 2002 until 2016 at our institution as recently published [9]. The study is derived from an analysis of the “Registry of Malignant Arrhythmia and Sudden Cardiac Death—Influence of Diagnostics and Interventions (RACE-IT)”, a single-center registry including consecutive patients presenting with ventricular tachyarrhythmias and aborted cardiac arrest being acutely admitted to the University Medical Center Mannheim (UMM), Germany, (clinicaltrials.gov identifier: NCT02982473) from 2002 until 2015. The study was carried out according to the principles of the Declaration of Helsinki and was approved by the medical ethics committee II of the Medical Faculty Mannheim, University of Heidelberg, Germany.

### 2.2. Inclusion and Exclusion Criteria

Consecutive patients with ventricular tachyarrhythmias were included [9]. The decision to treat patients with ACEi or ARB was based on the discretion of the cardiologists during routine care according to European guidelines [6,13,14,15]. Patients with death during index hospitalization, patients without ACEi or ARB treatment and patients with both ACEi plus ARB therapy were excluded from the present study. All other medical therapies apart from ACEi/ARB were allowed.

### 2.3. Primary and Secondary Endpoints

The follow-up period was set at three years for all outcomes. The primary prognostic endpoint was all-cause mortality. All-cause mortality was documented using our electronic hospital information system and by directly contacting state resident registration offices (Bureaux of Mortality Statistics) all across Germany. Identification of patients was verified by place of name, surname, day of birth and registered addresses. Secondary endpoints were a composite arrhythmic endpoint (i.e., recurrences of ventricular tachyarrhythmias, appropriate ICD therapies, sudden cardiac death) and cardiac rehospitalization. Cardiac rehospitalization comprised rehospitalization due to VT, VF, acute myocardial infarction (AMI), acute heart failure and inappropriate device therapy.

### 2.4. Statistical Methods

Quantitative data are presented as mean ± standard error of mean (SEM), median and interquartile range (IQR), and ranges depending on the distribution of the data and were compared using the Student’s *t*-test for normally distributed data or the Mann–Whitney U test for nonparametric data. Deviations from a Gaussian distribution were tested by the Kolmogorov–Smirnov test. Spearman’s rank correlation for nonparametric data was used to test univariate correlations. Qualitative data are presented as absolute and relative frequencies and compared using the Chi² test or the Fisher’s exact test, as appropriate.

Firstly, the univariable Kaplan–Meier method was applied to evaluate prognostic differences within the entire cohort. Then, the impact of ACEi versus ARB was analyzed separated by LVEF ≥ 35% and <35%. Thereafter, multivariable Cox regression models were developed using the “forward selection” option, where only statistically significant variables (*p* < 0.05) were included and analyzed simultaneously. Predefined variables being used for multivariable Cox-regressions included: baseline parameters (age, gender), chronic diseases (chronic kidney disease, diabetes mellitus), coronary artery disease (CAD), acute myocardial infarction (AMI), LVEF < 35%, the presence of an ICD and ACEi versus ARB therapy.

Secondly, propensity score matching was applied retrieving data from the entire patient cohort. In RCTs, patients have a 50% chance of being treated with or without a specific medication (such as ACEi or ARB). Balanced measured and unmeasured baseline characteristics would then be expected. In an observational study recruiting real-life patients, the specific treatment is not randomized, resulting in varying chances between 0% and 100% to receive it, including imbalances in baseline characteristics. Consequently, differences of outcomes in specific treatment groups might be explained by heterogenous distribution of baseline characteristics. However, the consecutive all-comer study reflects a realistic picture of current health-care supply. Therefore, to reduce this selection bias, we used 1:1 propensity scores for the receipt of a specific discharge medication (i.e., ACEi versus ARB) to assemble a matched cohort in which patients receiving and not receiving the discharge medication would be well balanced on all measured baseline characteristics. Propensity scores were created according to the presence of the following independent variables: age, sex, diabetes, CAD, LVEF, in-hospital CPR, out-of-hospital CPR, index ventricular tachyarrhythmias (i.e., VT/VF), chronic kidney disease, and the presence or absence of an ICD. Based on the propensity score values counted by logistic regressions, for each patient, one patient in the control group with a similar propensity score value was found (accepted difference of propensity score values < 5%). Thereafter, univariable stratification was performed using the Kaplan–Meier method with comparisons between groups using univariable hazard ratios (HR) given together with 95% confidence intervals. Propensity score matching was calculated within the entire study cohort and then separated by LVEF ≥ 35% and <35%.

The result of a statistical test was considered significant for *p* < 0.05. SAS, release 9.4 (SAS Institute Inc., Cary, NC, USA) and SPSS (Version 25, IBM, Armonk, NY, USA) were used for statistics.

## 3. Results

### 3.1. Study Population

**Figure 1:**
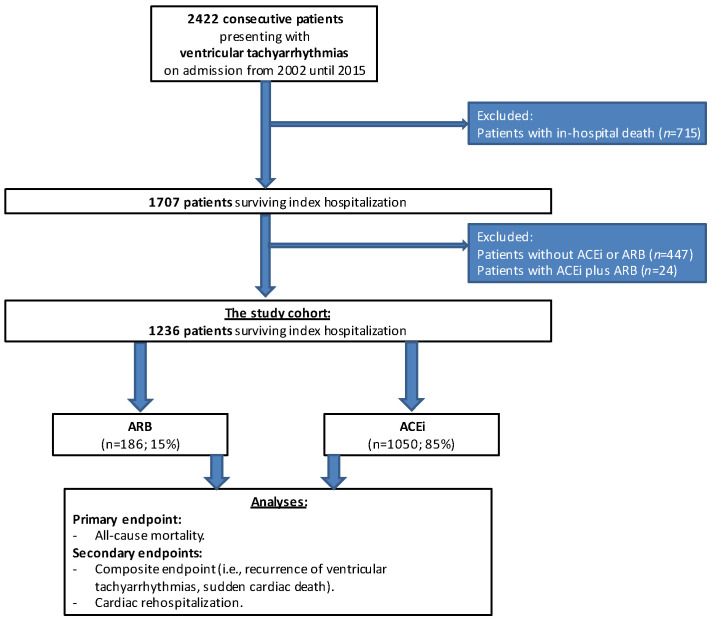
Flow chart of the study population.

From a total of 2422 patients with ventricular tachyarrhythmias, 715 were excluded for in-hospital death, 477 for receiving neither ACEi nor ARB treatment and 24 patients for receiving both ACEi and ARB therapy (Figure 1; flow chart).

The final study cohort comprised 1236 patients with ventricular tachyarrhythmias, 85% of whom were treated with ACEi and 15% were treated with ARB (*p* = 0.001). Within the ARB group, most patients were discharged on candesartan (53% with a mean daily dose of 15.3 mg ± 0.9 mg), followed by valsartan (19%; mean daily dose 121.0 mg ± 11.8 mg) (Table 1; study drugs). The most common type of ACEi was ramipril (71%; mean daily dose 5.4 mg ± 0.1 mg), whereas enalapril (19%; mean daily dose 12.3 mg ± 0.6 mg) and perindopril (5%; mean daily dose 3.5 mg ± 0.3 mg) were less common (Table 1).

As seen in Table 2 (left column), patients were median-aged at 69 years and most patients were males in both subgroups (75–77%). An index episode of VT was more common than VF in patients with ACEi and ARB treatment (66–73% vs. 27–34%; *p* = 0.087). In particular, the rates of arterial hypertension (79% vs. 65%; *p* = 0.001) and hyperlipidemia (42% vs. 34%; *p* = 0.047) were higher in patients treated with ARB. In contrast, rates of chronic kidney disease, prior heart failure and LVEF were equally distributed in both groups. Besides slightly higher rates of beta-blocker treatment in the ACEi group (89% vs. 83%; *p* = 0.032), no further differences regarding concomitant pharmacotherapies were observed.

### 3.2. Follow-Up Data, Primary and Secondary Endpoints within the Entire Study Cohort

**Figure 2:**
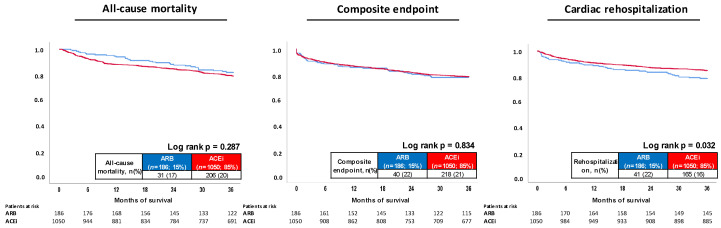
Prognostic impact of ACEi versus ARB treatment on all-cause mortality (**left panel**); risk of the composite arrhythmic endpoint (i.e., recurrence of ventricular tachyarrhythmias, sudden cardiac death) (**middle**); and cardiac rehospitalization (**right panel**) within the entire study.

Median follow-up time within the entire study cohort was 4.0 years (IQR 1.7–7.5 years). At three years of follow-up, the primary endpoint all-cause mortality occurred in 17% of the patients with ARB treatment and in 20% with ACEi. Accordingly, risk of all-cause mortality was not affected by treatment with ACEi versus ARB (log rank *p* = 0.287; HR = 0.965; 95% CI 0.689–1.351; *p* = 0.835) (Table 3 and Figure 2, left panel). Furthermore, risk of the composite endpoint was comparable in both groups (22% vs. 21%; HR = 1.227; 95% CI 0.841–1.790; *p* = 0.288). In contrast, ACEi was associated with a decreased risk of cardiac rehospitalization at three years (16% vs. 22%; log rank *p* = 0.032; HR = 0.690; 95% CI 0.490–0.971; *p* = 0.033) (Figure 2, middle and right panel).

### 3.3. Stratification by LVEF

**Figure 3:**
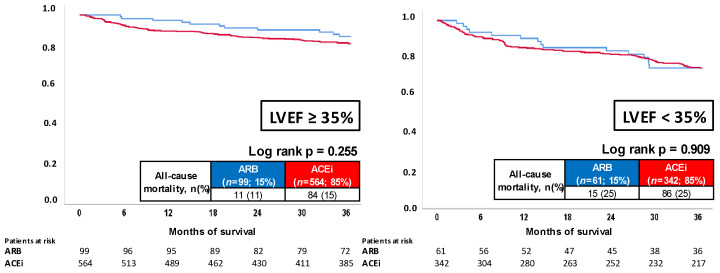
Prognostic impact of ACEi versus ARB treatment on all-cause mortality in patients with LVEF ≥ 35% (**left panel**) and LVEF < 35% (**right panel**).

Focusing on patients with LVEF ≥ 35%, no differences regarding all-cause mortality were observed in patients treated with ACEi or ARB (15% vs 11%; log rank *p* = 0.255; HR = 1.438; 95% CI 0.767–2.695; *p* = 0.258) (Figure 3, left panel). In line with those results, cardiac rehospitalization was not affected by ACEi or ARB (HR = 0.687; 95% CI 0.404–1.169; *p* = 0.166) (not shown).

In the presence of LVEF < 35%, similar mortality rates were observed at three years of follow-up (25% vs 25%; log rank *p* = 0.909; HR = 1.032; 95% CI 0.597–1.787; *p* = 0.909) (Figure 3, right panel), whereas a trend towards improved freedom from cardiac rehospitalization was seen in the ACEi group (HR = 0.624; 95% CI 0.385–1.012; *p* = 0.056) (not shown).

### 3.4. Multivariable Cox Regression Models

After multivariable adjustment, ACEi was not associated with an increased risk of all-cause mortality at three years compared to ARB therapy (HR = 1.457; 95% CI 0.952–2.229; *p* = 0.083) (Table 4). In contrast, increasing age (HR = 1.057; *p* = 0.001), presence of diabetes mellitus (HR = 1.654; *p* = 0.001), chronic kidney disease (HR = 1.489; *p* = 0.007) and LVEF < 35% (HR = 1.909; *p* = 0.001) were associated with impaired prognosis, whereas an ICD was associated with decreased long-term mortality (HR = 0.462; *p* = 0.001). In line with these results, the risk of the composite endpoint (HR = 1.028; 95% CI 0.717–1.475; *p* = 0.880) was not affected by ACEi/ARB. Finally, ACEi was associated with improved freedom from cardiac rehospitalization compared to ARB after multivariable adjustment (HR = 0.688; 95% CI 0.478–0.990; *p* = 0.044) (Table 4).

### 3.5. Propensity-Score Matched Cohorts

To re-evaluate the prognostic impact of ACEi versus ARB therapy in a more homogenous subgroup of patients, additional propensity score matching was performed. The characteristics of patients with ACEi and ARB therapy after propensity score matching are presented within Table 2 (right column). Following propensity score matching, especially age, sex, LVEF, chronic kidney disease and distribution of coronary artery disease were equally distributed among patients with ACEi or ARB therapy (Table 2, right column). In contrast, CPR was more common in patients with ACEi (25% vs. 18%; *p* = 0.048).

**Figure 4:**
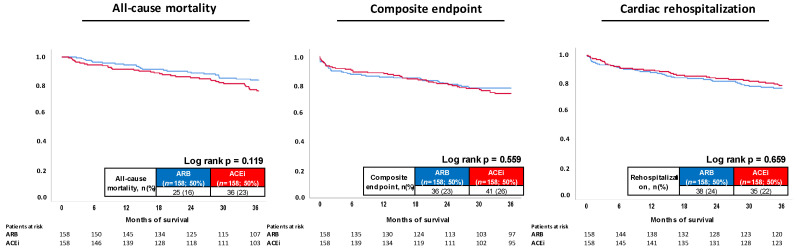
Prognostic impact of ACEi versus ARB treatment on all-cause mortality (**left panel**); risk of the composite endpoint (i.e., recurrence of ventricular tachyarrhythmias, sudden cardiac death) (**middle**); and cardiac rehospitalization (**right panel**) within the propensity-score matched cohort.

After propensity score matching, ACEi and ARB were associated with comparable prognosis regarding the primary endpoint of all-cause mortality (HR = 1.496; 95% CI 0.898–2.493; *p* = 0.122), as well as the secondary composite arrhythmic endpoint (HR = 1.142; 95% CI 0.730–1.787; *p* = 0.560) and cardiac rehospitalization (HR = 0.902; 95% CI 0.570–1.428; *p* = 0.660) (Figure 4).

**Figure 5:**
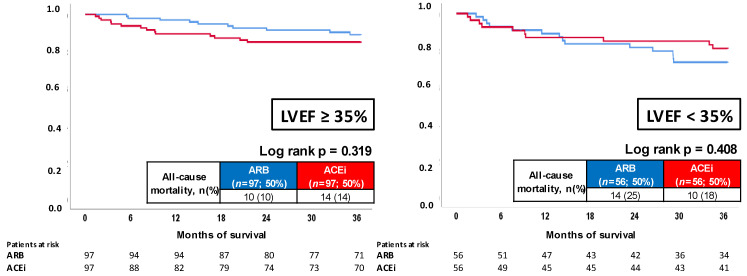
Prognostic impact of ACEi versus ARB treatment on all-cause mortality in patients with LVEF ≥ 35% (**left panel**); and LVEF < 35% (**right panel**) within propensity-score matched cohorts.

Thereafter, propensity-score analyses were performed in the subgroups of patients with LVEF ≥ 35% and <35%, respectively. In patients with LVEF ≥ 35% (*n* = 97 patients with ACEi and ARB), comparable all-cause mortality at three years was observed (10% vs. 14%; log ran *p* = 0.319; HR = 1.507; 95% CI 0.669–3.393; *p* = 0.322) (Figure 5, left panel). In line, the composite arrhythmic endpoint (HR = 1.734; 95% CI 0.848–3.547; *p* = 0.132) and cardiac rehospitalization (HR = 0.754; 95% CI 0.366–1.552; *p* = 0.443) were not affected by ACEi compared to ARB therapy (not shown). In patients with LVEF < 35%, the risk of all-cause mortality (15% vs. 25%; log rank *p* = 0.408; HR = 0.711; 95% CI 0.315–1.602; *p* = 0.410) (Figure 5, right panel), composite arrhythmic endpoint (HR = 0.572; 95% CI 0.298–1.096; *p* = 0.092) and cardiac rehospitalization (HR = 0.562; 95% CI 0.274–1.149; *p* = 0.114) was equally distributed among patients with ACEi or ABB therapy (not shown).

## 4. Discussion

The present study evaluates the prognostic impact of ACEi versus ARB treatment on the primary endpoint of all-cause mortality, as well as on secondary endpoints, such as a composite arrhythmic endpoint (i.e., recurrence of ventricular tachyarrhythmias, appropriate ICD therapies, SCD) and cardiac rehospitalization at three years in patients surviving index episodes of ventricular tachyarrhythmias.

This study suggests a comparable risk of all-cause mortality in patients treated with ACEi compared to ARB. ACEi and ARB had a comparable effect on the composite arrhythmic endpoint. Decreased risk of cardiac rehospitalization was no longer observed in patients treated with ACEi after propensity score matching.

The class I recommendation of ACEi/ARB for prevention of ventricular tachyarrhythmias relies on studies investigating the prognosis of ACEi/ARB in patients with HF and LVEF ≤ 40% for primary prevention of sudden cardiac death [6,8]. However, by preventing adverse cardiac remodeling, inhibitors of the renin angiotensin aldosterone system may also reduce the risk of arrhythmic events in patients with HF and AMI due to reduced cardiac fibrosis, lowering the risk of arrhythmic border zones [16]. In contrast to ACEi, ARB increases circulating angiotensin II levels by unopposed stimulation of the angiotensin II receptor, which increases plaque instability and the risk of thrombus formation [17].

However, real-life comparisons of ACEi and ARB are limited and mainly restricted to patients with AMI and systolic HF [6]. For instance, a recent meta-analysis including six randomized HF or AMI trials suggested a comparable risk of AMI, HF-related hospitalization, mortality, cardiovascular events and stroke in patients treated with ACEi as compared to ARB [18]. Furthermore, prognosis of patients treated with ACEi versus ARB was investigated within a study by Her et al., including over 13,000 patients with AMI undergoing percutaneous coronary intervention (PCI). At three years of follow-up, ACEi treatment was associated with a decreased risk of major adverse cardiac events (MACE), repeated revascularization and HF-related hospitalization when compared to ARB therapy [19]. In contrast, comparable risk of death, recurrent AMI, revascularization and risk of MACE was reported within a propensity-matched cohort including 3811 diabetics with ST-segment AMI at two years [20]. The present study, however, has a different point of view, including only patients with ventricular tachyarrhythmias, that have highest risk of death and recurrent arrhythmic events. No differences regarding all-cause mortality and the composite endpoint were observed, suggesting no additional benefit of ACEi regarding arrhythmic endpoints as compared to treatment with ARB. Due to the small number of patients with AMI in the present study (i.e., only 22 patients with ARB), further sub-analysis comparing ACEi and ARB were beyond the scope of the present study.

Focusing on patients without impaired LVEF, the prognostic role of ACEi and ARB was comprehensively investigated in 3006 patients with acute coronary syndrome and preserved ejection fraction (i.e., LVEF ≥ 40%). A comparable risk of all-cause mortality, as well as similar rates of the composite endpoint (i.e., death, AMI and HF) were demonstrated in patients treated with ACEi as compared to ARB [21]. These comparable effects may rely on the comparable effect of ACEi and ARB reducing the synthesis of angiotensin II, which represents a cornerstone in the pathogenesis of arrhythmic events on a structural, cellular and electrophysiological level [17].

In conclusion, the present study did not observe long-term differences in all-cause mortality in patients treated with ACEi or ARB.

### Study Limitations

This observational and retrospective registry-based analysis reflects a realistic picture of consecutive health-care supply of high-risk patients presenting with ventricular tachyarrhythmias. Pharmacological therapies were based on discharge medication at the index event. Changes in pharmacological treatment (i.e., discontinuation, dose adjustment) as well as side effects occurring during follow-up were not available for the present study. Furthermore, episodes of recurrent ventricular tachyarrhythmias, appropriate ICD therapies and cardiac rehospitalization were assessed at our institution only. Some remaining selection bias due to inhomogeneous distribution of baseline characteristics and comorbidities, as well as unmeasured cofounding among patients treated with ACEi or ARB may not be excluded despite multivariable Cox regression analyses and propensity score matching. The present results need to be re-evaluated within an even larger and more representative multi-center registry data or even RCT.

## Figures and Tables

**Table 1:** Study drugs.

Study Drugs; *n* (%); mg/day (Mean ± SEM)	ARB (*n* = 186; 15%)	ACEi (*n* = 1050; 85%)	*p* Value
Candesartan	99 (53)	-	-
	15.3 ± 0.9	-	-
Valsartan	36 (19)	-	-
	121.0 ± 11.8	-	-
Lorsartan	21 (11)	-	-
	53.8 ± 4.9	-	-
Other type of ARB	30 (16)	-	
Ramipril	-	740 (71)	-
	-	5.4 ± 0.1	-
Enalapril	-	195 (19)	-
	-	12.3 ± 0.6	-
Perindopril	-	12 (5)	-
	-	3.5 ± 0.3	-
Other type of ACEi	-	103 (10)	-

ACEi, angiotensin converting enzyme inhibitor; ARB, angiotensin receptor blocker; SEM, standard error of mean.

**Table 2:** Baseline characteristics before and after propensity score matching.

	Without Propensity Score Matching					With Propensity Score Matching				
Characteristic	ARB (*n* = 186; 15%)		ACEi (*n* = 1050; 85%)		*p* Value	ARB (*n* = 158; 50%)		ACEi (*n* = 158; 50%)		*p* Value
**Age**, median (range)	68 (32–89)		67 (15–94)		0.001	68 (32–89)		68 (25–85)		0.239
**Male gender**, *n* (%)	139	(75)	810	(77)	0.473	119	(75)	126	(80)	0.345
**Ventricular tachyarrhythmias at index**, *n* (%)										
Ventricular tachycardia	135	(73)	695	(66)	0.087	112	(71)	110	(70)	0.806
Ventricular fibrillation	51	(27)	355	(34)		46	(29)	48	(30)	
**Cardiovascular risk factors**, *n* (%)										
Arterial hypertension	147	(79)	679	(65)	**0.001**	127	(80)	101	(64)	**0.001**
Diabetes mellitus	48	(26)	284	(27)	0.725	41	(26)	50	(32)	0.264
Hyperlipidemia	78	(42)	361	(34)	**0.047**	68	(43)	63	(40)	0.568
Smoking	46	(25)	365	(35)	**0.007**	39	(25)	62	(39)	**0.006**
Cardiac family history	20	(11)	118	(11)	0.846	17	(11)	17	(11)	1.000
**Comorbidities at index stay**, *n* (%)										
Prior myocardial infarction	61	(33)	291	(28)	0.157	53	(34)	56	(35)	0.723
Prior coronary artery disease	104	(56)	567	(45)	**0.004**	93	(59)	92	(58)	0.909
Prior heart failure	60	(32)	289	(28)	0.186	53	(34)	59	(37)	0.480
Atrial fibrillation	63	(34)	334	(32)	0.799	55	(35)	59	(37)	0.639
Non-ischemic cardiomyopathy	13	(7)	91	(9)	0.448	13	(8)	17	(11)	0.443
Cardiopulmonary resuscitation	35	(19)	346	(33)	**0.001**	27	(17)	40	(25)	**0.048**
In hospital	18	(10)	129	(12)		14	(9)	29	(18)	
Out of hospital	17	(9)	217	(21)		13	(8)	11	(7)	
Chronic kidney disease	84	(46)	428	(41)	0.254	76	(48)	67	(42)	0.309
COPD/asthma	18	(10)	83	(8)	0.416	13	(8)	15	(10)	0.692
Coronary angiography, *n* (%)	121	(65)	758	(72)	**0.048**	107	(68)	124	(79)	**0.031**
No evidence of CAD	40	(33)	177	(23)	0.102	34	(32)	28	(23)	0.415
1-vessel disease	23	(19)	197	(26)		21	(20)	31	(25)	
2-vessel disease	27	(22)	174	(23)		23	(22)	31	(25)	
3-vessel disease	31	(26)	210	(28)		29	(27)	34	(27)	
Chronic total occlusion	25	(21)	151	(20)	0.850	22	(21)	32	(26)	0.348
Presence of CABG	22	(18)	107	(14)	0.241	21	(20)	27	(22)	0.688
PCI	31	(26)	342	(45)	**0.001**	28	(26)	36	(29)	0.628
Acute myocardial infarction	22	(12)	326	(31)	**0.001**	19	(12)	31	(29)	0.064
STEMI	8	(4)	123	(12)	**0.002**	8	(5)	14	(9)	0.185
NSTEMI	14	(8)	203	(19)	**0.001**	11	(7)	17	(11)	0.235
**LVEF**, *n* (%)										
>55%	49	(31)	231	(26)	0.228	48	(30)	29	(18)	0.092
54–45%	17	(11)	149	(16)		17	(11)	23	(15)	
44–35%	33	(21)	184	(20)		32	(20)	37	(23)	
<35%	61	(38)	342	(38)		61	(39)	69	(44)	
No evidence of LVEF	26	-	144	-	**-**	-	-	-	-	**-**
**Cardiac therapies at index**, *n* (%)										
Electrophysiological examination	78	(42)	330	(31)	**0.005**	66	(42)	55	(35)	0.203
VT ablation therapy	20	(11)	61	(6)	**0.012**	15	(10)	8	(5)	0.130
**Presence of an ICD**, *n* (%)	109	(59)	560	(53)	0.184	100	(63)	105	(67)	0.556
**Medication at discharge**, *n* (%)										
Beta-blocker	155	(83)	933	(89)	**0.032**	136	(86)	144	(91)	0.157
Statin	126	(68)	752	(72)	0.283	108	(68)	117	(74)	0.264
Amiodarone	26	(14)	176	(17)	0.344	24	(15)	24	(15)	1.000
Digitalis	29	(16)	136	(13)	0.329	29	(18)	25	(16)	0.550
Aldosterone antagonist	29	(16)	128	(12)	0.199	32	(20)	17	(11)	**0.020**

ACE, angiotensin converting enzyme; ARB, angiotensin receptor blocker; CABG, coronary artery bypass grafting; CAD, coronary artery disease; COPD, chronic obstructive pulmonary disease; LVEF, left ventricular ejection fraction; NSTEMI, non-ST-segment myocardial infarction; PCI, percutaneous coronary intervention; SEM, standard error of mean; STEMI, ST-segment MI; VT, ventricular tachycardia. Bold type indicates *p* < 0.05.

**Table 3:** Endpoints and follow-up data before and after propensity score matching.

	Without Propensity Score Matching					With Propensity Score Matching				
Characteristics	ARB (*n* = 186; 15%)		ACEi (*n* = 1050; 85%)		*p* Value	ARB (*n* = 158; 50%)		ACEi (*n* = 158; 50%)		*p* Value
**Primary endpoint**, *n* (%)										
All cause-mortality, at 36 months	31	(17)	206	(20)	0.346	25	(16)	36	(23)	0.117
**Secondary endpoints**, *n* (%)										
Cardiac rehospitalization, at 36 months	41	(22)	165	(16)	**0.033**	38	(24)	35	(22)	0.689
Composite Endpoint (recurrence of ventricular tachyarrhythmias, sudden cardiac death), at 36 months	40	(22)	218	(21)	0.818	36	(23)	41	(26)	0.512
**Follow up times**, *n* (%)										
Hospitalization total; days (median (IQR))	9 (5–17)		14 (8–23)		0.069	10 (5–17)		13 (9–22)		**0.015**
ICU time; days (median (IQR))	1 (0–5)		3 (0–8)		**0.001**	2 (0–5)		2 (0–5)		**0.004**
Follow-up; days (mean; median (range))	1910; 1630 (68–4912)		1894; 1744 (15–5106)		0.399	1976; 1682 (68–4912)		1856; 1706 (18–5089)		0.418

ACE, angiotensin converting enzyme; ARB, angiotensin receptor blocker; ICU, intensive care unit; IQR, interquartile range. Level of significance *p* ≤ 0.05. Bold type indicates *p* ≤ 0.05.

**Table 4:** Multivariable Cox regression analyses.

Endpoint	HR	95% CI	*p* Value
**Mortality**			
Age	1.057	1.040–1.073	**0.001**
Males	1.226	0.861–1.747	0.259
Diabetes	1.654	1.234–2.219	**0.001**
Chronic kidney disease	1.489	1.115–1.987	**0.007**
Acute myocardial infarction	0.628	0.424–0.932	**0.021**
Coronary artery disease	1.124	0.790–1.598	0.516
LVEF < 35%	1.909	1.407–2.590	**0.001**
Presence of ICD	0.462	0.336–0.636	**0.001**
ACEi versus ARB	1.457	0.952–2.229	0.083
**Composite endpoint**			
Age	1.006	0.994–1.019	0.310
Males	1.220	0.854–1.741	0.275
Diabetes	0.834	0.614–1.133	0.245
Chronic kidney disease	0.945	0.723–1.236	0.682
Acute myocardial infarction	0.961	0.647–1.428	0.843
Coronary artery disease	0.718	0.531–0.972	**0.032**
LVEF < 35%	1.142	0.870–1.499	0.338
Presence of ICD	7.752	4.829–12.445	**0.001**
ACEi versus ARB	1.028	0.717–1.475	0.880
**Rehospitalization**			
Age	1.006	0.992–1.020	0.423
Males	1.164	0.784–1.728	0.452
Diabetes	0.917	0.658–1.278	0.608
Chronic kidney disease	1.174	0.872–1.579	0.291
Acute myocardial infarction	1.246	0.841–1.845	0.273
Coronary artery disease	1.294	0.874–1.916	0.198
LVEF < 35%	1.442	1.058–1.965	**0.021**
Presence of ICD	3.057	2.045–4.571	**0.001**
ACEi versus ARB	0.688	0.478–0.990	**0.044**

ACE, angiotensin converting enzyme; ARB, angiotensin receptor blocker; CI; confidence interval; HR; hazard ratio; ICD; implantable cardioverter-defibrillator; LVEF, left ventricular ejection faction. Level of significance *p* < 0.05. Bold type indicates statistical significance.

## Data Availability

The datasets used and/or analyzed during the current study are available from the corresponding author on reasonable request.
